# MR Optimum: A web-based open-source tool for standardized signal-to-noise ratio evaluation in MRI

**DOI:** 10.1016/j.cmpbup.2026.100235

**Published:** 2026-02-05

**Authors:** Eros Montin, Xuan Thao Nguyen, Riccardo Lattanzi

**Affiliations:** a Center for Advanced Imaging Innovation and Research (CAI^2^R), New York University Grossman School of Medicine, NY, NY, USA; b Bernard and Irene Schwartz Center for Biomedical Imaging, New York University Grossman School of Medicine, NY, NY, USA

**Keywords:** MRI, Signal-to-noise ratio, Cloud computing, Web UI

## Abstract

Signal-to-noise ratio (SNR) is a key performance metric in magnetic resonance imaging (MRI) to evaluate pulse sequences, receive coils, and image reconstruction algorithms. A variety of methods have been proposed to estimate SNR. However, the lack of consistent and broadly available open-source implementations has been a challenge for reliable SNR comparisons in clinical and research settings. To address this gap, this work introduces MR Optimum, a cloud-native, open-source platform for standardized SNR analysis. MR Optimum integrates established SNR estimation techniques within a flexible, modular software architecture. A web-based user interface supports data upload, task configuration, cloud computations, and real-time results visualization. MR Optimum leverages serverless computing technologies (AWS Lambda and Fargate) to perform scalable, event-driven processing of MRI rawdata and allow users to calculate SNR using established methods: multiple replicas, pseudo multiple replicas, generalized pseudo multiple replicas, and analytic methods. Results include SNR maps, noise covariance and noise coefficient matrices, coil sensitivity profiles, and g factor maps. The web interface enables interactive visualization and histogram analysis based on regions of interest. Results can be exported in MATLAB, NIfTI, and JSON formats. By providing a unified computational environment, MR Optimum ensures reproducibility, and democratizes access to state-of-the-art SNR estimation, promoting multi-center harmonization and quality assurance.

## Introduction

Magnetic resonance imaging (MRI) is an essential diagnostic modality due to its unique ability to visualize soft tissues and physiological processes. The signal-to-noise ratio (SNR) is often used to describe MR image quality with respect to the background noise. SNR is a commonly used metric for evaluating radio frequency (RF) coil performance, pulse sequence designs, and image reconstruction algorithms. Unreliable SNR measurements may yield misleading conclusions, especially when validating advanced approaches in clinical protocols [1]. In recent years, SNR has also become increasingly important for Artificial Intelligence (AI)-based MRI applications [2]. For example, image SNR is associated with the accuracy of segmentation and classification models [3,4]. Furthermore, the noise distribution on an MR image can bias AI training, causing neural networks to learn noise patterns and artifacts rather than meaningful tissue information. Image SNR is equally vital for radiomics, where high-dimensional intensity, texture, and shape features depend on stable SNR conditions to ensure reproducibility and generalizability across scanners and patient populations [5–8].

Despite its importance, accurate SNR quantification remains challenging. Variability in acquisition protocols, reconstruction algorithms, and noise statistics complicates universal measurement standards. Conventional guidelines, such as those from the National Electrical Manufacturers Association (NEMA) [9], are designed primarily for single-channel coils and standardized phantoms, failing to account for complexities introduced by modern multi-channel coil configurations and advanced reconstruction algorithms like compressed sensing and deep learning.

Over the past few decades, numerous SNR estimation strategies have been introduced. The Multiple Replica (MREP) method is considered the gold standard, but it is time-consuming and unfeasible in vivo because it calculates voxel-level noise from hundreds of repeated acquisitions, which are assumed to yield identical images. Another approach is to estimate the noise from the difference image between two identical acquisitions, offering efficiency compared to using multiple replicas. However, also this method is sensitive to motion and the calculation is based on a region of interest (ROI), so it does not yield spatially resolved SNR maps as the MREP method [1]. The Pseudo Multiple Replica (PMR) method mimics the MREP method, but relies on a single acquisition and leverages Monte Carlo simulations to create a stack of pseudo replicas [10]. While accurate and applicable to any image reconstruction algorithm, the PMR approach imposes high computational demands and requires specialized software [11]. The Generalized Pseudo Multiple Replica (GPMR) framework [12] addresses the computational burden by implementing a more efficient approach for noise estimation in complex coil configurations. Specifically, GPMR utilizes a moving window technique to calculate voxel-wise noise statistics from a single image, thereby reducing the need for generating a large stack of pseudo replicas. Analytic methods (AM) that generate images in SNR units have also been proposed for linear multiple-coil image reconstruction approaches. Kellman and McVeigh [13] proposed an analytic approach to reconstruct images in SNR units for fully sampled k-space acquisitions reconstructed using Root-Sum-of-Squares (RSS) or B_1_-weighted coil combination, as well as for under-sampled SENSE parallel imaging reconstructions. An analytic expression for the g factor in the case of GRAPPA parallel imaging reconstructions was introduced by Breuer et al. [14]

Collectively, these methods offer a set of solutions to estimate SNR for any MRI acquisition. However, since they are not available as open-source software tools, the use of different in-house implementations has prevented standardized SNR estimations, which can lead to inaccurate reporting of the SNR in scientific publications and unreliable comparisons of the results between studies. This work aims to address this problem by introducing MR Optimum (pronounced “Mister Optimum”), a web-based open-source platform that enables users to estimate SNR using the popular methods described above, within a consistent framework that can be connected to cloud computing. An open-source software (LAB–QA2GO) [15] was recently proposed to standardize SNR measurements in quality assurance protocols. Such software estimates SNR using a time series of DICOM images from repeated phantom acquisitions following a method developed for quality assurance in multi-center functional MRI studies [16]. Note that this method, which will be implemented in future releases, does not provide an image in SNR units like all the SNR analysis methods currently implemented. Other software that perform DICOM-based SNR calculations as part of quality assurance protocols are provided together with calibration phantoms (e.g., https://qmri.com/product/qcal-mr-software/). However, these products are not open-source and have limited scopes.

MR Optimum is part of Cloud MR, an ongoing project aimed at developing a comprehensive open-source software platform to simulate all aspects of the MRI experiment. Unlike existing in-house implementations or standalone desktop tools that support limited options, MR Optimum combines a variety of validated algorithms for SNR estimation with a serverless architecture that ensures consistent numerical accuracy across deployment modes, while reducing runtimes through dynamic resource scaling. The same workflow can be executed via the web-based GUI, programmatic API, or local desktop environment, enabling seamless integration into diverse research and clinical infrastructures.

### Software architecture and implementation

**Fig. 1.**
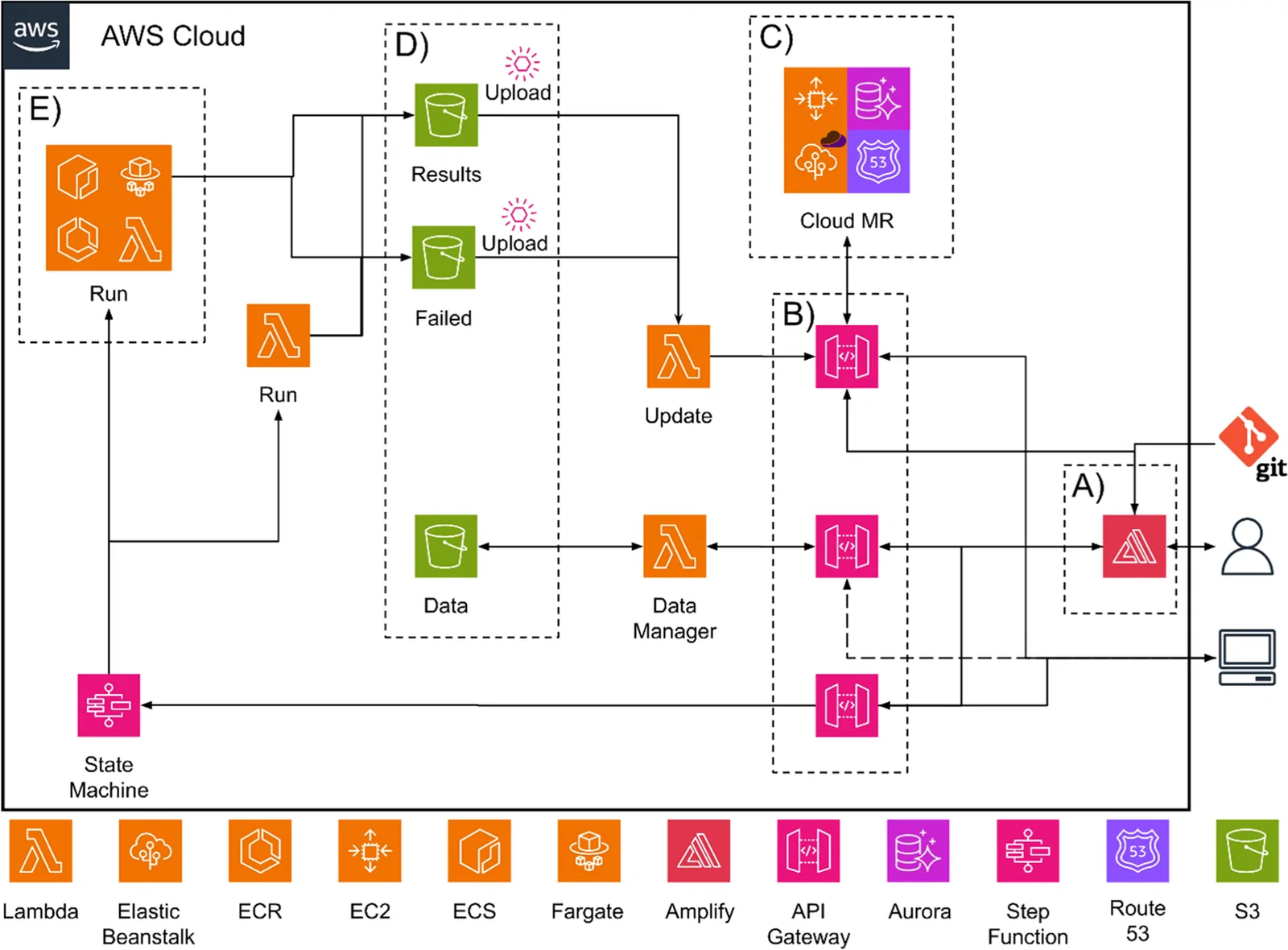
Schematic representation of the MR Optimum serverless software architecture. This diagram illustrates the cloud-based data flow and processing pipeline supporting user-driven SNR analysis. The “User” icon represents interaction through the web-based graphical interface, while the computer icon below it represents programmatic access to MR Optimum via RESTful APIs. Requests from the web GUI are handled by the Amplify-hosted frontend (dashed rectangular area A), where users upload the anonymized rawdata and configure the analysis parameters through the Data Manager module. RESTful API requests, represented by the computer icon, bypass the frontend and are sent directly to the API Gateway (dashed rectangular area B). API Gateway, an AWS service, authenticates incoming requests and routes them to the appropriate backend resources, enabling seamless communication between the frontend/API clients and the processing infrastructure Lightweight jobs are executed using AWS Lambda functions, while more computationally intensive tasks are delegated to AWS Fargate containers (dashed rectangular area E) running scalable, containerized workloads. Throughout the workflow, five Amazon S3 buckets: Job, Long Job, Data, Results, and Failed organize task configurations, intermediate files, and outputs. Backend status updates are managed via the Update Lambda (dashed rectangular area D), synchronizing progress with the application frontend and the main Cloud MR framework (dashed rectangular area C).

MR Optimum’s cloud-native software architecture (Fig. 1) is built on three layers: a user-friendly frontend, a serverless backend for high-performance computations, and a secure data management system for organizing user data and monitoring job status. This modular design simplifies scaling and provides flexibility towards establishing MR Optimum as a platform for both research and clinical MRI applications. MR Optimum operates within the Cloud MR framework (https://cmr.cloudmrhub.com), which handles user authentication, MRI data management, and distributed cloud-based task orchestration using a set of Application Programming Interfaces (APIs).

#### Web-based frontend

**Fig. 2.**
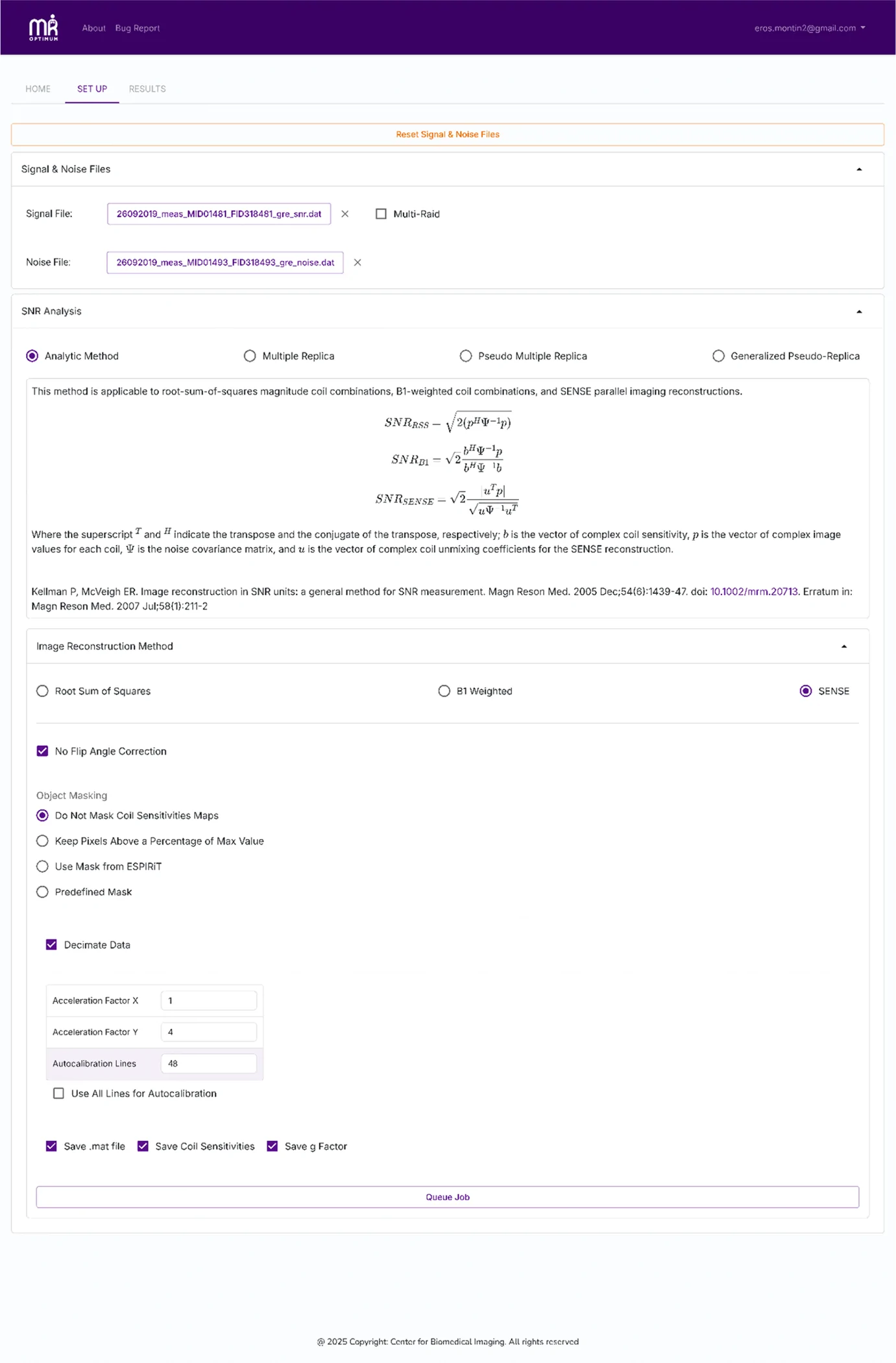
Screenshot of the web-based user interface. The MR Optimum web interface includes three main tabs: Home, Set Up, and Results. This screenshot shows the Set Up tab, where users first upload or select anonymized rawdata (signal and noise) and then configure the desired SNR analysis method and associated settings. The modular design allows users to queue jobs directly through the browser interface without requiring local installations or command-line interactions.

MR Optimum’s frontend (Fig. 2) was developed using React.js (https://react.dev/) and Redux (https://redux.js.org/) for workflow execution. It is deployed on AWS Amplify (Fig. 1A), a fully managed service that provides a streamlined framework for building, deploying, and hosting web applications. The web-based frontend also integrates Niivue’s WebGL-based visualization engine for real-time volumetric data rendering (https://niivue.com/).

The frontend consists of three tabs. The Home tab displays a table with the available data to the user. Users’ data and results are stored in isolated S3 storage paths keyed to their account and are inaccessible to other users, including in the case of multi-tenant deployments, unless explicitly shared. From the Set Up tab, users can create and queue the computational jobs. The Results tab lists the status of the submitted jobs and allows users to visualize and analyze the completed ones. The Set Up tab has multiple expanding panels. The first one enables users to upload anonymized rawdata (i.e., k-space samples) of the MR acquisition (“signal”) and the associated noise-only rawdata (“noise”), acquired with the same coil-sample configuration but with the transmit voltage turned off. The current implementation of MR Optimum is compatible with Siemens rawdata files, which, once uploaded, are stored in an Amazon Simple Storage Service (S3) cloud repository and can then be selected by the users from a dropdown menu. Rawdata can be anonymized by users while exporting them from the MR scanner console, or subsequently, using open-source tools, such as the *TwixMRIAnonymizer* in the case of Siemens rawdata.

MR Optimum currently supports only Cartesian acquisition schemes, but radial and spiral trajectories will be added in future versions, as well as the possibility of handling Magnetic Resonance Data (MRD) files, a vendor-neutral standard for representing MRI raw k-space data and associated metadata [17]. The desired SNR analysis (AM, MREP, PMR, GPMR) can be configured from the second panel of the Set Up tab. The user then chooses the image reconstruction method among the available ones: Root Sum of Squares (RSS) and matching filter B_1_-weighted (B1) coil combination, or Sensitivity Encoding (SENSE) and Generalized Autocalibrating Partially Parallel Acquisitions (GRAPPA) for parallel imaging. In the case of parallel imaging, users can either upload prospectively acquired undersampled k-space data or decimate k-space data to simulate acquisitions for different acceleration levels.

**Fig. 3.**
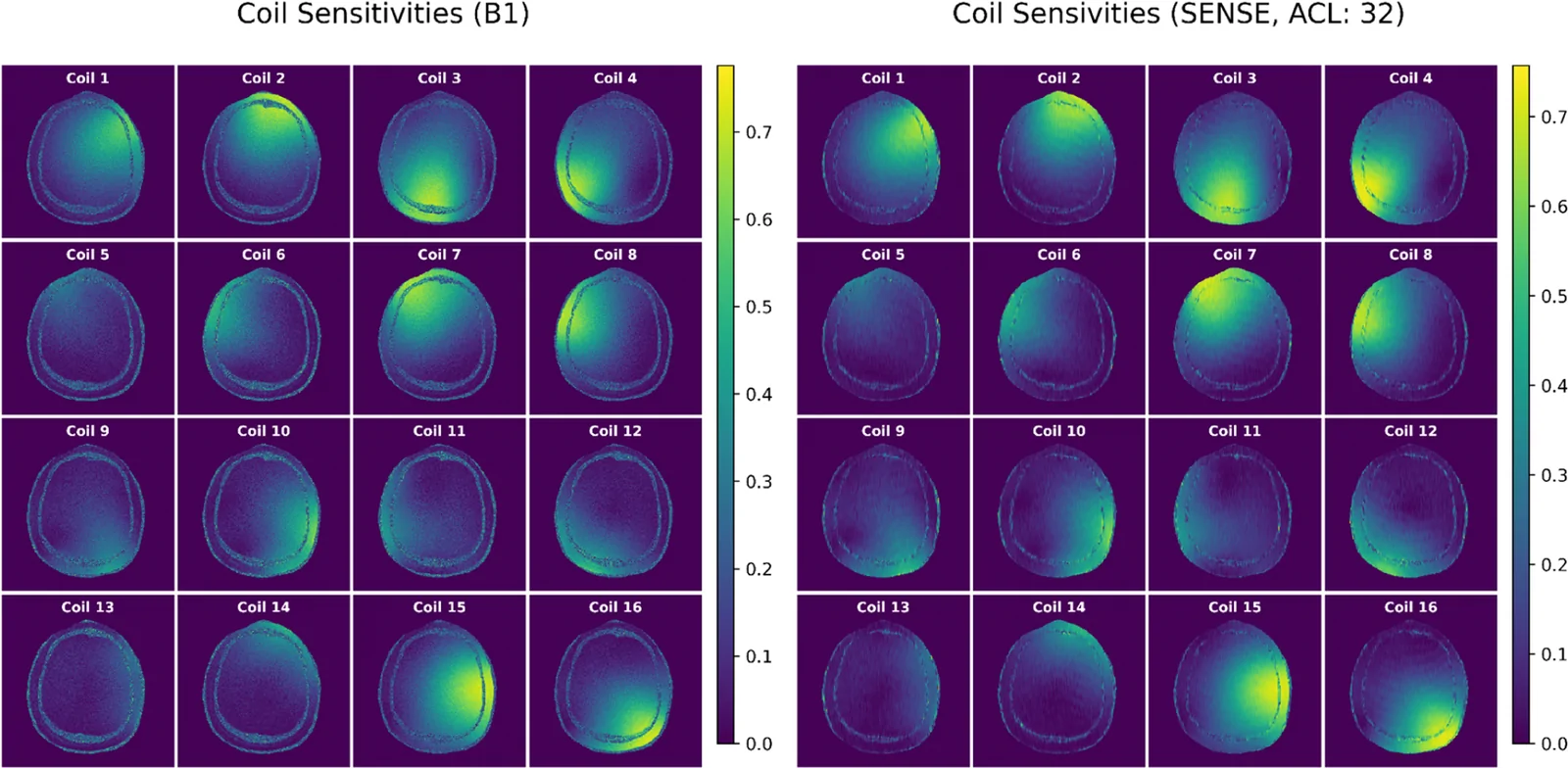
Representative coil sensitivity maps of a 16-channel 3 T receive array calculated within the B1 and SENSE SNR estimation methods. In B1 (left), coil sensitivities are computed using the fully sampled k-space data. In SENSE (right), coil sensitivities are estimated from the autocalibration lines (ACL = 32 in this example) of the undersampled k-space data. The latter show Fourier truncation artifacts due to using only the central lines of k-space.

Coil sensitivity maps are required for B1 and SENSE. Users can upload coil sensitivity maps or estimate them. MR Optimum estimates the coil sensitivities from the multi-coil k-space signal data after pre-whitening them with the noise covariance matrix calculated from the noise data. For B1, the sensitivities are computed from the fully sampled k-space data by reconstructing individual coil images and normalizing them by the RSS of all coil images. For SENSE, the maps are computed from the ACL, which are read from the rawdata in the case of prospective undersampling, or specified by the users in the case of retrospective undersampling. To ensure dimensional consistency with the full acquisition, the low-resolution ACL k-space is zero-padded along the phase-encoding direction (and, if necessary, the frequency dimension) to match the full matrix size before reconstruction. This avoids aliasing and allows the coil sensitivity maps to align spatially with the final reconstructed images. Since truncating the k-space to extract only the central ACL region introduces Gibbs ringing artifacts, a Hanning window (with width equal to the ACL region) is applied to the calibration k-space prior to inverse FFT reconstruction. This smooths the k-space edges, reducing oscillations in the image domain and improving the stability of the sensitivity estimation. The resulting coil images are then each normalized by the RSS combination of all of them to generate smooth, artifact-reduced sensitivity maps. Users have the option to mask the coil sensitivities to remove background noise and artifacts before using them for image reconstruction. An example of estimated coil sensitivity maps obtained using the B1 approach (from fully sampled data) and the SENSE approach (from ACL-based calibration) is shown in Fig. 3 for a representative brain dataset.

All calculations are executed within the MR Optimum backend using open-source implementations. RSS, B1, and SENSE reconstructions were implemented in-house and are included in the cloudmr-tools library (https://github.com/cloudmrhub/cloudmr-tools). GRAPPA was implemented using the pygrappa package. The current implementation of MR Optimum does not use vendor-specific reconstruction software, ensuring full reproducibility across environments and deployment modes. The classes for the AM, MREP, PMR, and GPMR analysis methods are available in the mroptimum-tools package (https://github.com/cloudmrhub/mroptimum-tools) and pseudocode is included in the supplementary material of this article.

Each queued job has a unique ID and an alias that can be edited by the user. The Results tab displays a table with all jobs and their status (pending, completed, failed). Users can click on a completed job to visualize the results. The visualization panel relies on Niivue’s rendering capabilities to display a volumetric SNR map and the noise covariance matrix. Depending on the SNR analysis, users can also visualize coil sensitivities and g factor maps. Comparative statistics and histograms of user-defined ROIs can be computed in real time. ROIs can be saved in an AWS S3 bucket and then imported when needed. Results can be exported as a single zip file, which includes MATLAB and NIfTI files of the data, a JSON log for reproducibility, and pre-generated scripts for offline analysis.

### Secure data management

The web-based frontend is integrated with the data management system of Cloud MR (Fig. 1C), developed using Laravel (https://laravel.com/, a PHP framework) and AWS Aurora (MySQL-compatible). This backend provides a structured repository for user accounts, MRI acquisition metadata, and real-time pipeline monitoring. Laravel’s native support for JSON Web Token (JWT) authentication ensures secure web and API communications by enforcing consistent session handling and access control. In addition to managing the core database operations, the Laravel backend also powers multiple RESTful APIs that are essential for the frontend’s functionality and service endpoints, maintaining data integrity and platform security.

To ensure scalability and maintainability of the overall platform, Cloud MR is deployed as a stand-alone web app using AWS Elastic Beanstalk, a fully managed service for application deployment that automatically handles infrastructure provisioning, load balancing, scaling, and monitoring. Domain Name System (DNS) resolution is managed through Amazon Route 53 (Fig. 1C), a scalable cloud DNS service that directs users’ requests to infrastructure endpoints, ensuring access to the https://cmr.cloudmrhub.com domain.

### Serverless backend processing

MR Optimum employs a hybrid architecture to orchestrate modular data workflows and dynamically scale computational resources based on task demands. For rapid computation, without persistent infrastructure, the system leverages AWS Lambda, a Function-as-a-Service (FaaS) solution that provides serverless computing by executing lightweight code functions in response to events, without provisioning or managing servers, making it ideal for short-duration tasks (Fig. 1). For more computationally intensive tasks, it utilizes AWS Fargate, a Container-as-a-Service (CaaS) platform that enables the serverless deployment of Docker containers, supporting scalable, isolated execution of workloads without the need for explicit server management (Fig. 1E). This hybrid approach enables MR Optimum to achieve optimal performance and cost-efficiency by dynamically allocating resources according to workload complexity, thereby minimizing operational overhead. Each execution instance, whether running on Lambda or within a Fargate container, operates in an isolated environment preconfigured with standard imaging libraries such as NumPy [18], SciPy [19], SimpleITK [20], ITK [21,22], and VTK [23]. These libraries support a broad spectrum of image processing algorithms, covering tasks from basic pre-processing to advanced volumetric reconstructions.

MR Optimum organizes data and computations using three dedicated buckets on Amazon S3: Data, Results, and Failed (Fig. 1D). This approach ensures that each stage of the pipeline, from user data to storing final outputs, remains distinct and traceable. Task orchestration is started by AWS API Gateway (Fig. 1A), a fully managed service that enables developers to create, publish, maintain, monitor, and secure APIs at any scale, facilitating seamless communication between the frontend and backend services. It authenticates incoming requests (using JWT credentials) and employs AWS Step Function that in the first state classifies jobs based on the computational resource they require. Tasks expected to complete in <15 min are routed to an AWS Lambda function, while more computationally demanding workflows (e.g., PMR calculations, datasets with multiple slices, etc.) are routed to an AWS Fargate containerized runtime for extended or iterative calculations. Both execution paths share the same codebase but are distinguished by their architectural configurations. Specifically, the Fargate deployment executes the computations using a container provisioned with 8 virtual CPUs and 16 GB of RAM, thereby supporting extended or iterative tasks with greater resource requirements. Throughout the process, the Data bucket maintains the rawdata inputs and the intermediate data (coil sensitivity maps, ROIs, noise covariance matrices), while the results of successfully executed computations are transferred to the Results bucket. Should a job fail, logs and diagnostic files are routed to the Failed bucket, facilitating quick troubleshooting and error resolution. Upon completion, whether successful or not, results and logs are updated in real time by the Update Lambda function, which checks the appropriate S3 bucket and synchronizes the job status with the frontend using Cloud MR APIs. In this way, users receive immediate notifications on task progress, outcomes, or errors.

#### MR optimum interaction workflow

**Fig. 4.**
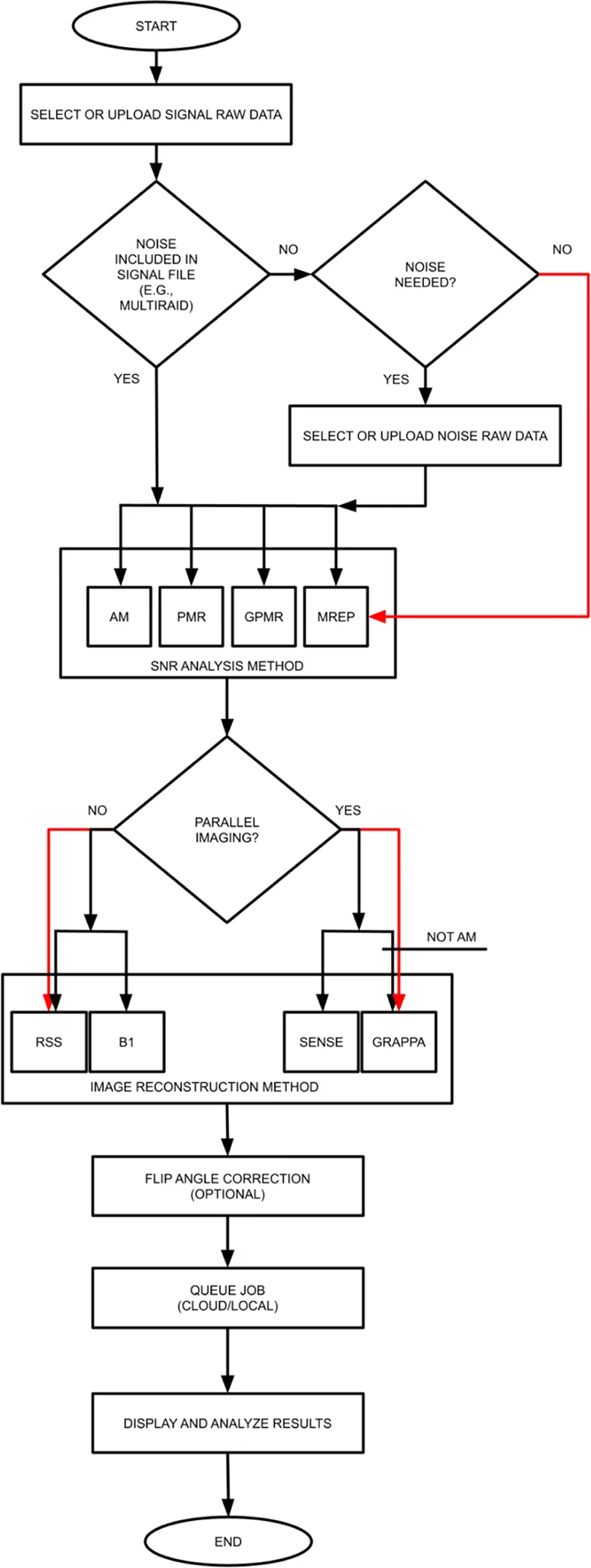
SNR analysis workflow in MR Optimum. Users first upload a rawdata file with the MR signal. If the noise data is not included in the signal file but it is needed for the SNR analysis, then users upload a separate noise rawdata file (acquired by turning off the transmit voltage). Then, they select the desired SNR analysis method. Next, users choose an image reconstruction method. In the current implementation, Root Sum of Squares (RSS) and B1-weighted combination (B1) are available for fully sampled data, while SENSE and GRAPPA are available for parallel imaging reconstructions, using either prospectively undersampled or retrospectively decimated data. Note that if separate noise data are not available, then only the multiple replica method (MREP) with RSS or GRAPPA reconstruction is available (red arrows paths). Also note that, in the case of AM, the GRAPPA reconstruction only provides the g factor, since an analytic expression for the SNR is not available. Users have the option to upload a flip angle map to correct for transmit field inhomogeneities. The configured pipeline is then queued as a new job and processed either on cloud-based resources or local environments. When the calculation is complete, results are synchronized to the frontend and users can display and analyze the generated maps.

**Fig. 5.**
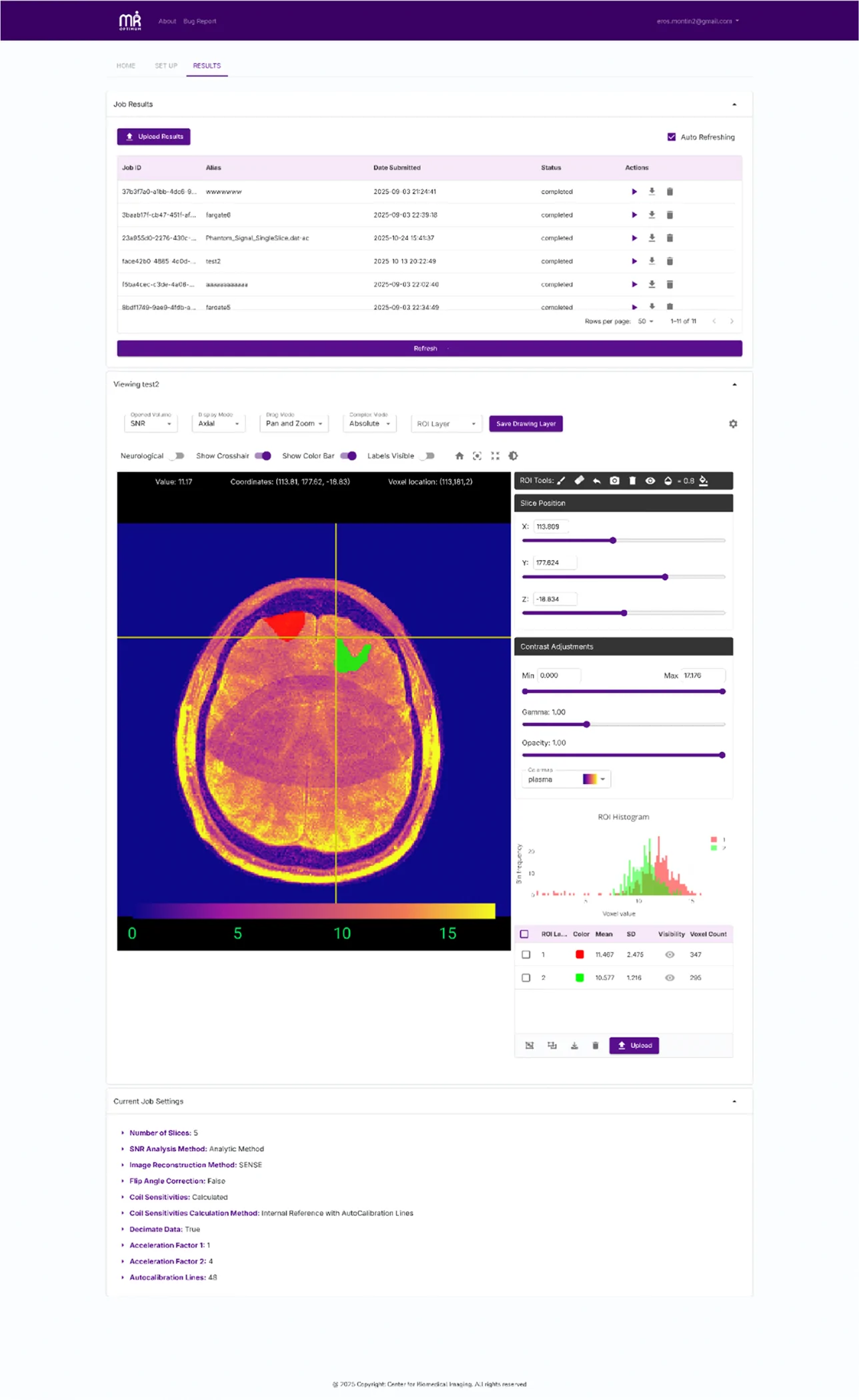
Screenshot of the Results tab. The Results tab enables users to visualize and analyze the outcome of completed jobs. This screenshot shows the SNR map for a representative axial slice through the center of the brain, estimated using the Analytic Method for a four-fold accelerated SENSE reconstruction. Two user-defined regions of interest (ROIs) are shown and highlight differences in SNR between a frontal region, closer to the receive coils, and a more central region of the brain. Real-time statistics and histograms of selected ROIs are displayed below the visualization panel, providing an intuitive assessment of image quality and encoding performance within anatomically or clinically relevant zones.The reduced SNR observed in central regions reflects spatially varying g-factor–induced noise amplification inherent to parallel imaging acceleration.

MR Optimum provides an intuitive, structured workflow for seamless user interaction and automated data processing, clearly illustrated in Fig. 4. After uploading a rawdata of interest or selecting from a list of previously uploaded rawdata, users must select the SNR analysis method (Fig. 2) among AM, MREP, PMR or GPMR. User-submitted tasks, including rawdata uploading, are securely routed by the AWS API Gateway (Fig. 1B), which authenticates and classifies each request based on computational requirements. Users then specify an image reconstruction method and simply queue the new task. If noise rawdata are not available, users can only use the MREP method, with either RSS coil combination or GRAPPA parallel imaging reconstruction. In all other cases, users can currently choose between RSS and B1 to reconstruct fully sampled k-space data, or between SENSE and GRAPPA to reconstruct undersampled k-space data. Users have the option to retrospectively decimate k-space data to simulate different acceleration factors for parallel imaging. After setting up the task calculation, users interact with the results through the Results tab of the application interface (Fig. 5). From this tab, users can visualize the SNR map, the coil sensitivity profiles, the g factor, and the inverse g factor maps. The GUI supports interactive drawing of ROIs, enabling the extraction of summary statistics and real-time histogram evaluation of selected metrics across the ROI. Users can also upload previously saved ROIs or external ones generated by other software, provided they are in NIfTI format. By enabling users to upload ROIs, MR Optimum makes it easy to use the same ROIs across different SNR estimation settings or to benchmark external segmentations. Each result component can be selectively visualized, compared, or exported, offering flexibility in both qualitative inspection and quantitative assessment. This structured, event-driven architecture efficiently handles complex SNR workflows, abstracting computational complexities and promoting scalable, reproducible, and collaborative MRI research, accessible from any internet-connected device.

### Deployment architecture

MR Optimum is designed with a flexible and modular deployment strategy, supporting three primary modes of execution to accommodate diverse computational needs and data governance requirements. The Python implementations of the four SNR estimation methods and the image reconstruction algorithms currently supported by MR Optimum will be made available after publication at https://cmr.cloudmrhub.com/apps/mroptimum/.

### Mode 1: Cloud execution via a shared infrastructure

In its default configuration, MR Optimum operates as a cloud-native platform, with all computations executed on pre-configured AWS clusters hosted by Cloud MR. Users can access the platform through the dedicated web-based interface at https://mro.aws.cloudmrhub.com or via its RESTful API. This shared-service model simplifies access and onboarding, since users do not have to install anything and can schedule an SNR calculation directly from the web browser. Job results can be downloaded via dedicated endpoints that direct to a zip file in the Results S3 Bucket (Fig. 1), allowing for seamless integration into downstream processing pipelines.

### Mode 2: User-deployed cloud infrastructure

Users not associated with the Cloud MR project can deploy MR Optimum on their own AWS account using the AWS Serverless Application Model (SAM) (https://aws.amazon.com/serverless/sam/). Deployment templates and configuration instructions are available through the public repository at https://github.com/cloudmrhub/mroptimum-app, enabling full control over execution resources, data locality and regionality, and usage constraints. This mode provides users with unrestricted computational throughput while preserving all the scalability and automation benefits of a serverless architecture. Users can instantiate the entire application stack (Fig. 1), including AWS Lambda functions, S3 storage, API Gateway interfaces, and an Amplify-hosted frontend, via SAM using a few simple commands. Similarly to Mode 1, users will then access the platform via the web-based graphical interface, which is deployed within their Virtual Private Cloud (VPC) using AWS Amplify, ensuring that all data processing and user interactions remain confined within their secure cloud environment.

### Mode 3: Local execution on desktop environments

MR Optimum also supports a fully local execution mode, ideal for environments with strict data privacy requirements or limited internet access. A dedicated Google Colab notebook guides users through environment configuration, dependency installation (e.g., NumPy, SciPy, ITK, SimpleITK), and execution of SNR estimation routines (https://github.com/cloudmrhub/mroptimum-tools/blob/main/mroptimum_tools.ipynb). The local version shares the same Python code that runs in the backend of the cloud implementation, ensuring numerical consistency across platforms. With support for multi-threaded execution and vectorized operations, computations are efficient even on standard workstations. Additionally, users can batch process large datasets, integrate custom models, or tailor pipelines to specialized research applications.

## Discussion

MR Optimum aims at addressing the longstanding challenges in the standardization of SNR evaluation in MRI, laying the groundwork for improved reproducibility in clinical research by integrating multiple SNR estimation strategies into a unified framework. The event-driven architecture enables nearly-instantaneous scaling and parallel processing. MR Optimum dynamically allocates computational resources, preserving stable runtimes despite surges in workload or data volume. This adaptability could be invaluable for collaborative research consortia that rely on streamlined, standardized SNR assessments.

In addition to providing browser-based accessibility, MR Optimum offers improved computational efficiency and reproducibility compared to typical offline SNR calculation scripts. By leveraging serverless execution, the platform minimizes runtime for demanding methods such as PMR and GPMR without compromising accuracy. The same SNR estimation and image reconstruction algorithms implemented in the cloud backend, including AM, MREP, PMR, and GPMR together with RSS, B1-weighted, SENSE, and GRAPPA reconstructions, can be executed using APIs on local workstations, facilitating integration with multi-site studies regardless of hardware or operating system constraints. MR Optimum enables users to perform reliable SNR assessments without programming experience, computational overhead, or data storage limitations, which could facilitate adoption by clinicians and imaging scientists.

The methods currently integrated in MR Optimum were previously validated with a phantom using MREP with 100 replicas as the gold standard SNR estimation [11]. The study found 6 % mean relative deviation for AM, 7 % for PMR, and 10 % for GPMR with respect to the SNR maps calculated with MREP. The availability of popular image reconstruction methods facilitates the standardization of SNR reporting, which is particularly valuable in multi-center studies and clinical trials. In future releases, thanks to the modular architecture, the MREP, PMR and GPMR methods in MR Optimum could be extended in a straightforward manner to include other image reconstruction algorithms, for example, to help benchmark new techniques during development. The MR vendors could also integrate the SNR estimation methods in MR Optimum at the scanner console, to generate images in SNR units for their proprietary image reconstruction software. Incorporating SNR monitoring within the MRI clinical workflow, by embedding PMR directly into the image reconstruction pipeline at the scanner could allow MR operators to adjust acquisition parameters and optimize image quality based on accurate and reproducible metrics.

The capabilities of MR Optimum could be further expanded with the integration of AI. For example, AI-driven noise modeling could enable adaptive noise correction that accounts for spatial heterogeneities and scanner-specific artifacts [24].

The current version of MR Optimum is limited to Siemens rawdata files. Future releases will rely on the MRD format [17], which will require the implementation of open-source tools to convert proprietary vendor rawdata into the MRD format. Similar tools are already available but are not consistently maintained and are compatible only with a limited number of scanner manufacturers and file formats. The rawdata conversion tools in future iterations will also include support for radial and spiral k-space trajectories, since the current version of MR Optimum can only process data from Cartesian acquisitions.

A practical limitation relates to the deployment infrastructure. While MR Optimum is fully open source, the reference web-based deployment relies on commercial cloud services to enable elastic scaling, parallel execution, and reproducible runtime environments. Use of such cloud infrastructure may incur usage-based costs that depend on workload size, storage requirements, and execution frequency. This may limit adoption. Note, however, that the software tools are not tied to a specific cloud provider: the same algorithms and APIs can be executed on local workstations or high-performance computing systems, enabling cost-free use beyond existing hardware resources.

Another limitation is that processing time and error diagnostics are not displayed directly in the user interface. Both are recorded in execution logs that can be downloaded as part of the result file generated for each executed job. However, the result file is not generated, and the log cannot be accessed if a job fails. To address this, the next release will enable users to directly visualize the log file in the frontend, which will provide error feedback. Note that a real time progress bar or an estimate of the processing time cannot be accurately implemented because the serverless cloud computing architecture dynamically allocates resources for computation. Output verification is not yet implemented. Future updates will add automatic checksum generation, further strengthening reproducibility and user experience.

We also plan to introduce automated reporting tools that generate publication-ready visualizations, statistical analyses, and metadata-driven quality assessments. The addition of structured, exportable reports would facilitate the integration of MR Optimum into routine quality assurance protocols in clinical and research settings. Finally, optimizing the allocation of cloud resources remains a priority to ensure sustained performance and scalability as the platform evolves. Further refinements in computational task orchestration, latency reduction for real-time data processing, and improvements in error recovery mechanisms will contribute to an even more robust user experience.

To our knowledge, MR Optimum is the first open-source web-based tool that includes multiple SNR estimation methods that operate directly on rawdata, integrating both analytic and Monte Carlo–based calculations within a unified platform. Future versions of MR Optimum will include additional SNR estimation methods that operate on reconstructed MR images (e.g., DICOM files), for example, the difference image method [1], the extension of the analytic methods in [13] for the case of magnitude images, or other methods normally used for MRI quality assurance protocols [16].

The adoption of a web-based interface reflects a broader trend in digital health systems, where browser-accessible platforms are increasingly used to facilitate real-time interaction, data visualization, and user engagement across distributed environments. This paradigm not only lowers the entry barrier for users with diverse technical backgrounds but also enhances the accessibility of advanced computational tools within both clinical and research contexts [25]. The combination of a serverless modular architecture and flexible deployment options further democratizes access to the state-of-the-art SNR calculation methods available in MR Optimum and enhances standardization in SNR reporting.

## Supplementary Material

1

Supplementary material associated with this article can be found, in the online version, at doi:10.1016/j.cmpbup.2026.100235.
