# Supplementary material for: MR Optimum: A web-based open-source tool for standardized signal-to-noise ratio evaluation in MRI
Source: Comput Methods Programs Biomed Update. Author manuscript; Available in PMC 2026 Aug 20. (PMC13489594; doi:10.1016/j.cmpbup.2026.100235)
Supplement: Supplementary file 1 [file NIHMS2186362-supplement-1.docx]

Multiple Replicas pseudocode

Compute the SNR by reconstructing several *real* replicas (identical scans acquired multiple times) and measuring voxel-wise variability.

Steps:

1. Initialize an empty imageArray = []

2. For each replica r in range(R):

a. Set reconstructor.SignalKSpace = signalKSpace[..., r]

b. If reconstructor.HasSensitivity:

reconstructor.ReferenceKSpace = corresponding ref

c. Run image = reconstructor.getOutput()

d. Append image to imageArray

3. Compute:

Mean(x, y) = mean(|imageArray(x, y, :)|)

Std(x, y) = std(|imageArray(x, y, :)|, ddof=1)

4. Compute voxel-wise SNR:

SNR(x, y) = Mean(x, y) / Std(x, y)

Output:

SNR map (2D image)

Pseudo Multiple Replicas pseudocode

Estimate SNR when only one acquisition is available, by **simulating multiple noisy versions** of the k-space using the measured noise covariance.

Steps:

1. Compute correlation factor:

corr = get_correlation_factor(noiseCovariance)

2. Retrieve signal k-space and, if applicable, the acceleration pattern:

S = reconstructor.getSignalKSpace()

if reconstructor.HasAcceleration:

accel_pattern = reconstructor.getAccelerationPattern2D()

else:

accel_pattern = None

3. Generate pseudo replicas by injecting correlated noise:

For i in range(numberOfReplicas):

a. Create synthetic complex Gaussian noise N ~ CN(0, Rn)

scaled by corr to preserve coil correlation

b. If accelerated:

N = N * accel_pattern

c. K_noisy = S + N

d. reconstructor.setSignalKSpace(K_noisy)

e. image_i = reconstructor.getOutput()

f. Add image_i to imageArray

4. If no reference image exists:

referenceImage = reconstructor.getOutput()	# reconstruction without added noise

5. Compute the SNR denominator D:

a. max_val = max(abs(imageArray))

b. D(x, y) = std( abs(imageArray(x, y, :)) + max_val, axis=-1, ddof=1 )

c. Replace any D ≤ ε with 1 to avoid division by zero

d. Cache D for reuse

6. Compute voxel-wise SNR:

SNR(x, y) = referenceImage(x, y) / D(x, y)

Output:

SNR map (2D image)

Generalized Pseudo Multiple Replicas pseudocode

Enhance pseudo multiple replica SNR estimation using pixel neighbor information and replicas to estimate the noise component of the SNR.

Steps:

1. Generate pseudo replicas as before → imageArray

2. Compute mean image:

MeanImage = mean(|imageArray| across replicas)

3. Get reference (noise-free) image:

RefImage = reconstructor.getOutput()

4. Compute local residual map:

Residual(x, y) = RefImage(x, y) - MeanImage(x, y)

5. Estimate spatial noise power using Wiener filter:

NoiseMap = get_wien_noise_image(Residual, boxSize)

\# Typically: local variance in a moving window

6. Compute SNR:

SNR(x, y) = RefImage(x, y) / NoiseMap(x, y)

Output:

Spatially adaptive SNR map

Supplementary Figure 1: Example of RSS reconstruction

Supplementary Figure 2: example of a 16 channel Noise Covariance Matrix

Supplementary Figure 3: example of a 16 Sensitivity Maps for a dataset SENSE with acceleration set to 1 x 4 and 48 ACL lines

Supplementary Figure 4: example of SNR map or a dataset SENSE with acceleration set to 1x4 and 48 ACL lines

Supplementary Figure 5: example of G factor map for a dataset SENSE with acceleration set to 1x4 and 48 ACL lines

Supplementary Figure 6: example of inverse G factor map for a dataset SENSE with acceleration set to 1x4 and 48 ACL lines
